# Statement of Retraction: Fischer's oligopeptide ratio in ischemic hypoxia: prophylactic amendment of sophoretin and melatonin supplementation

**DOI:** 10.1080/20565623.2026.2745389

**Published:** 2026-10-16

We, the Editor and Publisher of the Journal ***Future Science OA***, have retracted the following article:

**O Kadry, M., & Ali, H. M. (2024). Fischer’s oligopeptide ratio in ischemic hypoxia: prophylactic amendment of sophoretin and melatonin supplementation. *Future Science OA*, *10*(1).** https://doi.org/10.2144/fsoa-2023-0117

After publication, concerns were raised by a third party in 2025 regarding the integrity of figure 3E in the article.

Further investigation by the Journal and Publisher confirm unusual overlaps within the individual comet assay image panels in Figures 3B and 3C.

When approached for an explanation, the authors have provided their original data. However, the integrity concerns in Figures 3B and 3C remain unaddressed, and further integrity concerns were identified in the original data sent.

As the editorial team no longer have confidence in the reported conclusions, we are retracting the article. The corresponding author listed in this publication has been informed. The author does not agree with the retraction.

We have been informed in our decision-making by our editorial policies and the COPE guidelines. The retracted articles will remain online to maintain the scholarly record, but they will be digitally watermarked on each page as ‘Retracted’.

